# The Effect of Omega-3 Fatty Acids on Thromboxane, Brain-Derived Neurotrophic Factor, Homocysteine, and Vitamin D in Depressive Children and Adolescents: Randomized Controlled Trial

**DOI:** 10.3390/nu13041095

**Published:** 2021-03-27

**Authors:** Zuzana Paduchová, Barbora Katrenčíková, Magdaléna Vaváková, Lucia Laubertová, Zuzana Nagyová, Iveta Garaiova, Zdenka Ďuračková, Jana Trebatická

**Affiliations:** 1 Faculty of Medicine, Institute of Medical Chemistry, Biochemistry and Clinical Biochemistry, Comenius University, Sasinkova 2, 813 72 Bratislava, Slovakia; zuzana.paduchova@fmed.uniba.sk (Z.P.); katrencikov2@uniba.sk (B.K.); magdalena.vavakova@med.lu.se (M.V.); lucia.laubertova@fmed.uniba.sk (L.L.); 2 Juvenalia, s.r.o, Pediatric Centre, Veľkoblahovská 44A, 929 01 Dunajská Streda, Slovakia; juvenaliads@gmail.com; 3 Research and Development Department, Cultech Ltd., Unit 2 Christchurch Road, Port Talbot SA12 7BZ, UK; ivetag@cultech.co.uk; 4 Department of Paediatric Psychiatry, Faculty of Medicine, The National Institute of Children’s Diseases, Comenius University, Limbová 1, 833 40 Bratislava, Slovakia; jana.trebaticka@fmed.uniba.sk

**Keywords:** depressive disorder, omega-3 fatty acids, thromboxane, brain-derived neurotrophic factor, homocysteine, vitamin D, children and adolescents

## Abstract

In the DEPOXIN project, we have found that a high ratio of omega-6/omega-3 fatty acids (FA) is associated with worsening of depressive symptoms in children and adolescents with depressive disorder (DD) and that the 12-week omega-3 FA supplementation modulates DD symptoms. Here we present our results of the secondary outcomes: the levels of thromboxane (TXB), brain-derived neurotrophic factor (BDNF), homocysteine (HCy) and vitamin D. Fifty-eight patients were randomized into two arms. One group received a fish oil emulsion enriched with omega-3 FA, and the other received a sunflower oil emulsion containing omega-6 FA, for 12 weeks. Depressive symptoms were evaluated, using the Child’s Depressive Inventory (CDI). The patients with DD had elevated TXB levels and decreased vitamin D levels, as compared to healthy controls. Both CDI and omega-6/omega-3 ratio correlated positively with TXB and negatively with BDNF at baseline. Compared to the omega-6 FA group, the supplementation with omega-3 FA for 12 weeks significantly reduced plasma TXB (*p* = 0.024) and increased BDNF (*p* = 0.011) levels. No changes in HCy and vitamin D were observed. Our results demonstrate the possible role of TXB and BDNF in the pathophysiology of DD and the benefits of omega-3 FA supplementation. The study was registered with the ISRCTN registry (ISRCTN81655012).

## 1. Introduction

Depressive disorder (DD) is a serious global issue in children and adolescents, with a prevalence of about 5.7% among those aged 13–18 years old [1,2].

Core symptoms for childhood depression include persistent sadness, loss of interest and enjoyment, and increased fatigability [3]. The pathophysiology of DD in children and adolescents is not fully understood [4,5].

Over the years, the diet ratio of omega-6 fatty acids (FA) to omega-3 FA has changed from 1:1 to 15–20:1. [6]. These findings led to the hypothesis that omega-3 FA supplementation could be a way to treat DD [7,8,9]. Such an assumption is supported by information on the omega-3 FA biological effects (formation of anti-inflammatory metabolites, their effect on fluidity of cell membranes modulating membrane functions, and regulation of gene expression) [10,11]. The relationship between mental health and inflammation, as well as increased levels of markers of inflammation, was found not only in adults but also in children and adolescents with DD [12]. However, information on the relationship between the depression severity and markers of inflammation is insufficient in young individuals, due to the lack of studies and their heterogeneity [13]. In meta-analyses of 22 studies with children and adolescents, Colasanto et al. [14] demonstrated that depression positively correlates with inflammation represented by C-reactive protein (CRP) or interleukin IL-6.

Polyunsaturated fatty acids are involved in the regulation of inflammation and fluidity of membranes and may contribute to the pathophysiology of DD. Arachidonic acid (AA, C20:4, ω = 6), which is formed by elongation/desaturation of linoleic acid (LA, C18:2, ω = 6), is a substrate for the synthesis of eicosanoids, such as prostacyclins, thromboxanes, and prostaglandins (e.g., PGF2 and PGE2), stimulating proinflammatory cytokine synthesis. The eicosanoids are formed by the catalytic action of cyclooxygenase (COX). On the other hand, omega-3 fatty acids, such as eicosapentaenoic acid (EPA, C20:5, ω = 3) and docosahexaenoic acid (DHA, C22:6, ω = 3), are metabolized by COX and lipoxygenase to polyhydroxy metabolites, e.g., D-resolvins formed from DHA, or to trihydroxy metabolites, e.g., E-resolvins formed from EPA, both with anti-inflammatory properties. For these reasons, the ratio between omega-6 and omega-3 FA plays an important role in the regulation of inflammatory homeostasis [15]. An increased omega-6/omega-3 FA ratio leads to the displacement of DHA from its structures in the brain by arachidonic acid, leading to an increase in the turnover of AA to pro-inflammatory eicosanoids [16].

In our recent work [9], we demonstrated that children with depressive disorder have a higher omega-6/omega-3 ratio compared to healthy controls (24.23/1 vs. 19.3/1, *p* = 0.017), and after three months of omega-3 FA supplementation, this ratio dropped to 7.6/1 in DD patients.

Several studies have examined plasma/urine levels of thromboxane B (TXB), to determine the pro-inflammatory platelet activation as an indirect marker of inflammation in the body [17,18]. It was found that a high omega-6/omega-3 ratio increases platelet aggregation and TXB2 formation [19]. Some studies have shown the higher TXB levels in depressed individuals or in adult patients with bipolar disorder [18,20], while others have not confirmed this observation [21]. In children and adolescents, there is a lack of information on the relationship between the thromboxane level and the severity of depression.

Brain-derived neurotrophic factor (BDNF) is one of the growth factors, neurotrophins. Deficiency in neurotrophins in adults may contribute to hippocampal pathology during the development of depression. BDNF regulates a key transcription factor that is involved in neuroprotection, neuroinflammation, and cellular growth [22]. However, there are controversial reports about the effects of BDNF on depression [23]. Pandey et al. [24] have found decreased gene and BDNF protein expression in lymphocytes, as well as decreased BDNF levels in the platelets of adult and pediatric depressed patients. Sen et al. and Yasui-Furukori et al. [25,26] also reported reduced BDNF levels in the serum of depressed individuals, compared to healthy controls. However, an elevated level of serum BDNF levels was found in depressed patients but did not correlate with the severity of depression or suicidality [27]. Gender differences were found in the production of BDNF in the serum of depressed adolescents. The lower BDNF levels were observed in females, compared to adolescent males and healthy controls [28]. The association of omega-3 FA with BDNF level can be explained by the activation of BDNF transcription by the DHA molecule through the activation of the PI3K/Akt signaling pathway [29]. However, the decreased serum DHA concentrations were found in pediatric and adolescent patients with DD, when compared to controls, whereas the BDNF level was not reduced in DD patients [28].

Evidence suggests that homocysteine (HCy) level is associated with depression, vascular diseases, and disorder of neurotransmission in adults. In adults, an association between the higher concentration of HCy and increased risk of depression was found. It was calculated that the reduction of HCy by 0.19 mg/L could decrease depression by about 20% [30]. Little information on HCy’s involvement in DD pathology in children and adolescents is available. The increased HCy levels have been observed in depressed adolescents, compared to the controls [31]. Chung et al. [32] found higher serum HCy levels in boys (12–13 years old) with moderate-to-high anxiety levels. However, no association was observed between serum HCy levels and depression in both boys and girls.

Vitamin D is known to be important for the calcium and phosphorous metabolism and bone health, but it is also assumed to have an effect on mental health [33]. Vitamin D is known to transcriptionally activate tryptophan hydroxylase 2, which forms serotonin from tryptophan [34] and modulates the hypothalamic–pituitary–adrenal axis, regulating catecholamine production through vitamin D receptors [35]. In addition, EPA is able influence the release of serotonin from presynaptic neurons by decreasing prostaglandin PG2 levels, and DHA can modify serotonin receptors through modulation of membrane fluidity in neurons [36]. A causal relationship between vitamin D and depressive symptoms in adults is not expected [37,38]. Following the vitamin D supplementation, an improvement in depressive symptoms assessed by parents was observed in adolescent patients with the mild depression and low vitamin D levels (<12 ng/mL), but not in self-rated depression [39]. A negative association between vitamin D levels and severity of depression has been found in depressed adolescents [31].

In our DEPOXIN project (Molecular basis of depression disorder and influence of omega-3 fatty acid on clinical symptoms and biomarkers), we have demonstrated the effect of fish oil emulsion rich in omega-3 FA on depressive symptoms in children and adolescents with DD (the primary outcomes) [9,40]. The aim of our project was to find the relationship of plasma thromboxane B2, brain-derived neurotrophic factor, homocysteine, and serum vitamin D levels with the depression severity, the omega-6/omega-3 FA ratio, serum EPA, and DHA concentrations in depressed children and adolescents (the secondary outcomes), as well as their comparison with healthy controls.

Our results should contribute to the understanding of the interrelationships between the observed parameters in children and adolescents with depressive disorder.

## 2. Methods

Subjects, inclusion and exclusion criteria, study design and intervention, randomization, patient enrollment, and treatment have been described in detail in our previous works [9,40].

Briefly, we enrolled thirty-one outpatients with depressive disorder (DD) and twenty-nine outpatients with mixed anxiety and depressive disorder (MADD) in our study (hereinafter collectively referred DD + MADD as DD. Data were analyzed from 58 patients who completed the 12-week intervention) (Supplementary Materials Figure S1).

Twenty healthy children and adolescents were enrolled to the healthy control group.

Patients were randomized into two arms (ratio 1:1) and received daily 20 mL of either fish oil emulsion rich in omega-3 FA (Om3 group) or a comparator of sunflower oil emulsion rich in omega-6 FA (Om6 group) for 12 weeks, alongside their standard antidepressant therapy (selective serotonin reuptake inhibitors; SSRI). The fish oil emulsion comprised 2400 mg of total omega-3 FA (1000 mg of EPA and 750 mg of DHA, EPA:DHA ratio = 1.33:1), and the sunflower oil emulsion contained 2467 mg of omega-6 linoleic acid. Both emulsions were prepared by Cultech Ltd., Port Talbot, UK.

Patients were advised to follow their standard diet and report any changes in their dietary habits throughout the study. The self-rated scale of Children’s Depression Inventory (CDI) was used to rate the depression severity [41,42]. More serious depressive state is represented by a higher CDI score.

### 2.1. Biochemical Parameters

Patients and healthy controls provided the venous blood samples after a 12 h overnight fast. The blood samples were centrifuged (1200× *g*, 10 min) within 1 h of collection, and the obtained serum and EDTA-plasma were stored at −80 °C, until further analysis.

Serum fatty acids were determined as methyl esters and separated by gas chromatography. A detailed procedure is described in our most recent previous work [9]. Omega-6/omega-3 ratio was calculated from (AA + LA)/(EPA + DHA) concentrations in μg/0.1 mL.

Thromboxane B2 in plasma was determined with a Thromboxane B2 EIA kit (Cayman Chemicals, No. 501020, Ann Arbor, Michigan 48108, MI, USA), according to the manufacturer’s protocol. The concentration of thromboxane is presented in pg/mL.

BDNF was determined in plasma with a Human BDNF ELISA kit (Sigma-Aldrich, No. RAB0026, St. Louis, MO, USA), according to the manufacturer’s protocol, and is expressed in ng/mL.

Homocysteine was determined in plasma by Advia Centaur XP HCZ kit (Siemens, Ref 09087913, USA) in the Advia Centaur system (Siemens Healthcare Diagnostics, Tarrytown, NY, USA). The homocysteine level is expressed in µmol/L.

Serum vitamin D was determined by a 25-OH Vitamin D total Elisa kit (Access 2 Immunoassay system, Beckman Coulter, Inc., Brea, CA, USA). The vitamin D concentration is expressed in ng/mL.

### 2.2. Statistical Analysis

Data are presented as the mean ± standard deviation (SD) for the normally distributed variables or as a median and interquartile range for data showing departures from normality. Student’s *t*-test or Wilcoxon signed-rank test were used to compare data between weeks of intervention, depending on the normality of the data. A non-parametric Mann–Whitney U test was used for the evaluation of difference between patients and healthy controls. The effect of omega-3 fatty acids on the studied parameters following 12 weeks of supplementation was evaluated with the two-way ANOVA test. The Spearman rank correlation coefficient was used for the correlation determination.

A value of *p* < 0.05 was considered statistically significant. Data analyses were performed, using StatsDirect^®^ 3.3.4 (StatsDirect Ltd., Birkenhead, Merseyside CH42 8NQ, UK) and IBM SPSS Statistics 25.0.0. The StatsDirect^®^ program was used for graphical representation of the data.

## 3. Results

The results of the effect of omega-3 FA on the depressive disorder symptom severity (the study primary outcome) were published by Trebatická et al. [9]. Briefly, a significant reduction in CDI scores was observed in the Om3 group after six weeks (−6.3 CDI score, –22%) and 12 weeks (−6.5 CDI score, –24%), compared to the Om6 group. In the Om3 group, the ratio of omega-6/omega-3 was reduced from 24.2/1 to 7.6/1 (–69%) after six weeks and to 9.93/1 (–60%) after 12 weeks. No changes were found in the Om6 group.

Basal serum levels of EPA (1.08 ± 0.61 µg/0.1 mL) and DHA (2.53 ± 1.02 µg/0.1 mL) were significantly lower (*p* < 0.001 for both), compared to the healthy controls (1.94 ± 0.8 and 3.74 ± 1.1 µg/0.1 mL, respectively). Following the omega-3 FA supplementation, EPA and DHA levels increased to 462% and 270% of baseline [9].

At baseline, the levels of EPA and omega-6/omega-3 ratio correlated with symptoms’ severity [9].

### 3.1. Baseline Data

The baseline characteristics of study participants already published [9,40] are presented in Table 1.

We analyzed the correlation among depressive symptoms rated as a CDI score (details are published in Trebatická et al., 2020 [9]) and BMI in all patients at the baseline. There was no significant relationship between CDI and BMI (*r* = 0.071, *p* = 0.297) in either depressed males (*r* = −0.116, *p* = 0.36) or depressed females (*r* = 0.133, *p* = 0.116). However, a positive correlation was found between BMI and the serum omega-6/omega-3 FA ratio in depressed patients (*r* = 0.244, *p* = 0.039), but not in the control group (*r* = −0.240, *p* = 0.335).

The plasma TXB, BDNF, HCy, and serum vitamin D levels at the baseline are shown in Table 2. The highly increased levels of TXB and reduced levels of vitamin D were observed in the patient group, compared to the healthy controls at the baseline (*p* < 0.001 and *p* = 0.031, respectively). We evaluated possible differences in basal levels of BDNF, CDI, omega-6/omega-3, EPA, and DHA between patients already treated and initially diagnosed, and no significant difference was found for any parameter. There was no significant difference in the BDNF and HCy levels between all patients and healthy controls.

**Figure 1:**
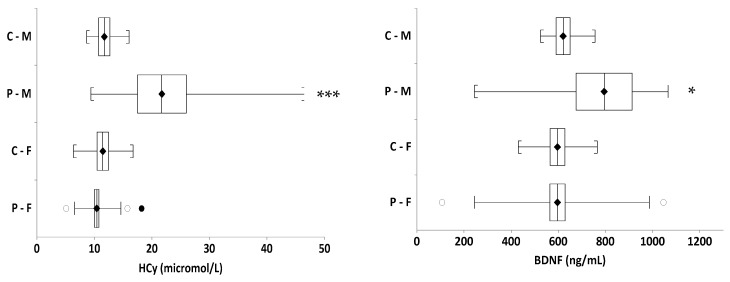
Levels of brain-derived neurotrophic factor (BDNF) and homocysteine (HCy), by gender, at the baseline. C = controls, P = patients, M = male, F = female; • outer fence for outlier, ° inner fence for outlier, * *p* < 0.05, *** *p* < 0001.

However, we found strong gender differences in BDNF and HCy levels at the baseline (Figure 1). Depressed boys had significantly higher BDNF levels (794.55 ± 376 ng/mL) and HCy levels (21.73 ± 14.1 μmol/L), compared to depressed girls at the baseline (BDNF: 596.41 ± 207 ng/mL, *p* = 0.027 and HCY: 10.4 ± 2.7 μmol/L, *p* < 0.0001). The statistically significant gender differences in BDNF and HCy levels were not observed in the healthy controls.

### 3.2. Effect of FA Supplementation in Patients with DD

The effect of omega-3 FA supplementation on the studied parameters is shown in Table 3.

**Figure 2:**
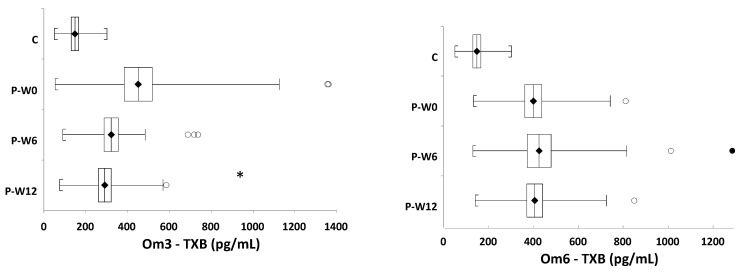
The levels of TXB in healthy controls and in patients with depressive disorder, during the intervention. C = controls, P = patients, Om3 = omega-3 patient intervention group, Om6 = omega-6 patient intervention group, TXB = thromboxane B, W = week of the intervention; • outer fence for outlier, ° inner fence for outlier, * *p* < 0.05 with respect to W0.

**Figure 3:**
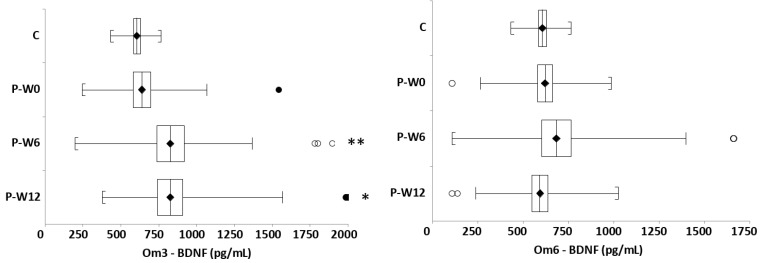
The levels of BDNF in healthy controls and in patients with depressive disorder, during the intervention. C = controls, P = patients, Om3 = omega-3 patient intervention group, Om6 = omega-6 patient intervention group, BDNF = brain-derived neurotrophic factor, W = week of the intervention; • outer fence for outlier, ° inner fence for outlier. * *p* < 0.05 with respect to W0, ** *p* < 0.01 with respect to W0.

The levels of TXB were reduced at week six, to 77.3% of baseline (*p* = 0.194), and at week 12 of supplementation, to 70.9% of baseline (*p* = 0.0374). Compared to baseline (100%), TXB values reached 100.3% and 98.7% (not significant, ns), following omega-6 FA administration after 6 and 12 weeks (Table 3, Figure 2). However, BDNF levels increased in the Om3 group, in contrast to the Om6 group, after six weeks to 130.9% of baseline (*p* = 0.009) and after 12 weeks to 129.9% of baseline (*p* = 0.04) (Table 3 and Figure 3).

Significant differences in TXB (*p* = 0.024) and BDNF (*p* = 0.011) levels were observed between Om3 and Om6 groups after 12 weeks of supplementation (Table 3). No significant changes in HCy and vitamin D levels were observed.

A significant treatment effect of omega-3 FA, compared to omega-6 FA, with two-way ANOVA, in BDNF, was confirmed after 12 weeks of intervention (*p* = 0.021). The TXB showed only a borderline significant treatment effect (*p* = 0.091).

### 3.3. The Correlations between Parameters

**Figure 4:**
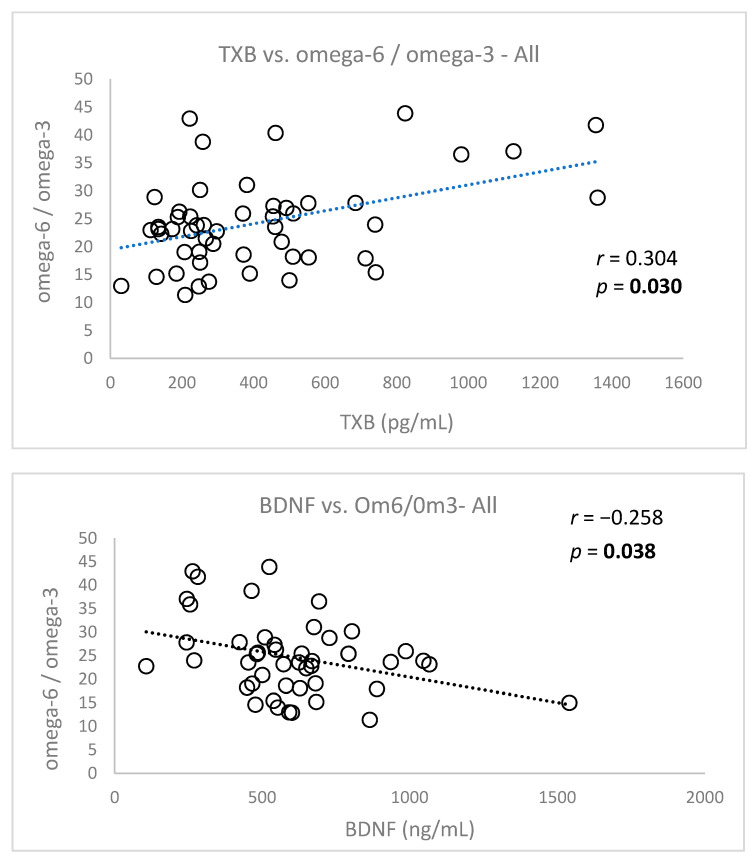
Correlations of basal TXB and BDNF values with omega-6/omega-3 ratio in patients with DD. TXB = thromboxane, BDNF = brain-derived neurotrophic factor, *r* = Spearman’s rank correlation coefficient, *p* = statistical significance.

The correlations between severity of depression (CDI score) or omega-6/omega-3 ratio and investigated parameters for patients with DD or healthy controls at baseline are presented in Table 4. The correlations between omega-6/omega-3 ratio and TXB or BDNF in depressed patients at baseline are presented in Figure 4.

**Figure 5:**
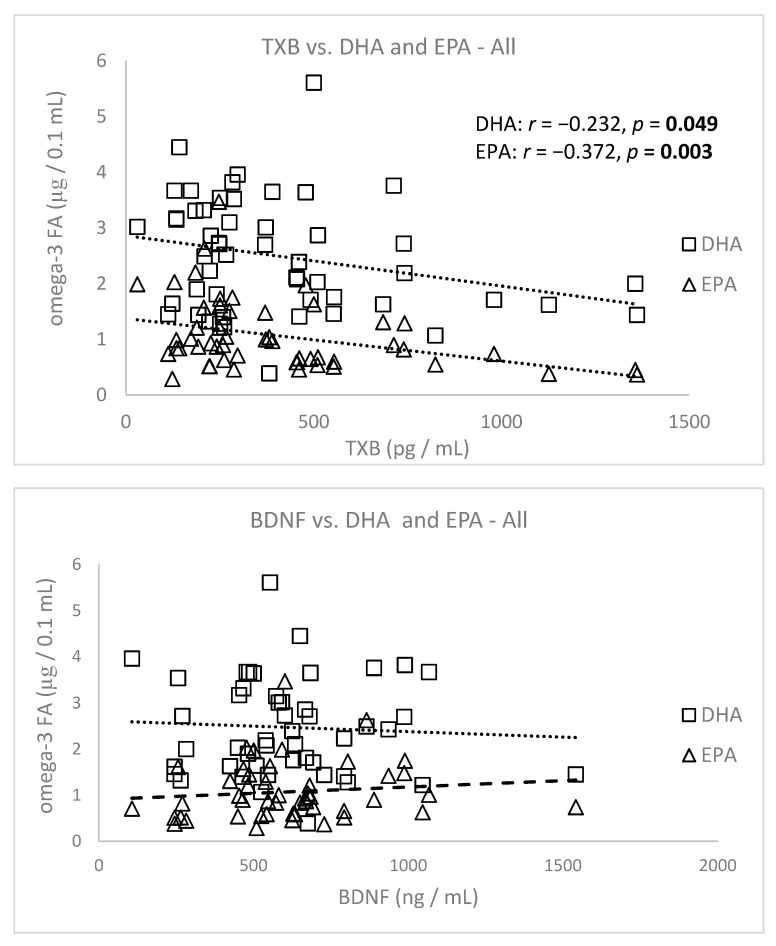
Correlations of basal TXB and BDNF with docosahexaenoic acid (DHA) and eicosapentaenoic acid (EPA) in depressed patients. TXB–thromboxane, BDNF–brain derived neurotrophic factor.

We evaluated the correlations of DHA and EPA levels with TXB and BDNF in all patients (FA levels were published in detail in our prior work [9]) (Figure 5). Surprisingly, BDNF did not correlate significantly with DHA or EPA, in contrast to TXB.

**Figure 6:**
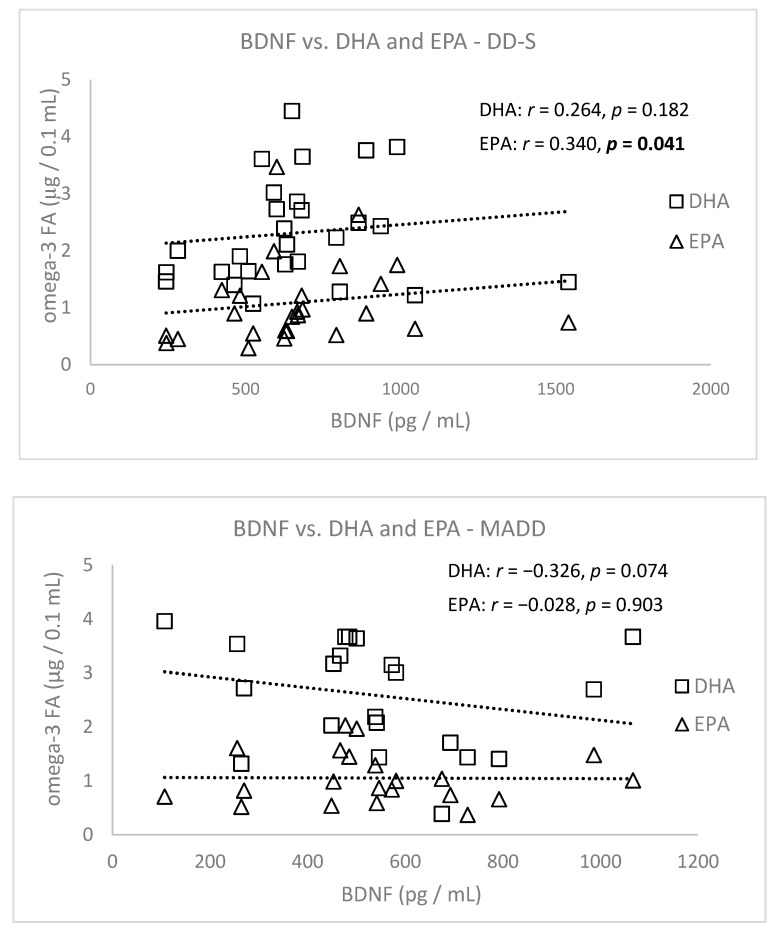
Correlations of BDNF with DHA and EPA in patients from the depressive disorder subgroup (DD-S, without mixed anxiety and depressive disorder (MADD)) and the MADD group. BDNF = brain derived neurotrophic factor, DHA = docosahexaenoic acid, EPA = eicosapentaenoic acid.

After dividing patients according to diagnoses into the depressive disorder subgroup (DD-S, *n* = 30) (DD without MADD) and mixed anxiety and depressive disorder (MADD, *n* = 25), only EPA but not DHA correlated positively with BDNF in patients in the DD subgroup. Patients with MADD did not show a correlation between these parameters (Figure 6). 

Following the 12-week supplementation with omega-3 or omega-6 FA, we did not observe any significant correlations between CDI or omega-6/omega-3 ratio and TXB, BDNF, HCy, or vitamin D in patients with DD (data not shown).

## 4. Discussion

The primary outcome of the DEPOXIN project was to investigate the effect of omega-3 FA on the severity of depression as determined by the CDI score, compared to the effect of omega-6 FA. We were the first to present a relationship between the omega-6/omega-3 FA ratio and the depression severity in children and adolescents [9].

We found in DD patients a positive correlation between BMI and omega-6/omega-3 ratio, but not between BMI and CDI. This contradicts the findings in a Swedish study where the authors found that obesity is associated with a risk of anxiety and depression independent of other factors (neuropsychiatric disorders and low socioeconomic status of the family) [43]. However, it should be noted that, in our group of depressed patients, 74.6% were normal weight, 23.7% were overweight, and only 1.7% were obese.

The secondary outcome of this trial was the comparison of the levels of thromboxane, brain-derived neurotrophic factor, homocysteine, and vitamin D in the pediatric population with DD and healthy controls, as well as the investigation of the effect of the omega-3 FA supplementation alongside the standard antidepressant therapy on depressive symptoms. A significant increase of TXB levels by 299% was observed in the group of depressed children and adolescents, compared to healthy controls (*p* < 0.001) independent of gender.

Thromboxane B2 (TXB2) is a marker of platelet activation. It is formed from AA under catalytic activity of cyclooxygenase (COX) and could be considered as an indirect marker of activated inflammatory pathways [20].

Consistent with the conclusions of Colasanto et al. [14], TXB levels significantly correlated with depressive symptoms in our patients, evaluated as the CDI score. Previously, we have demonstrated a significant correlation of the omega-6/omega-3 FA ration with CDI scores [9]. Similarly, the current results confirmed a significant positive correlation between TXB and omega-6/omega-3 FA ratio (*p* = 0.03) in the same patient cohort. These findings support the conclusions of DiNicolantonio and OKeefe [44], that an inflammatory response of the organism plays an important role in the pathophysiology of DD in children. Moreover, our previously published results [9,40] suggest that the omega-6/omega-3 FA ratio plays a role in the pathophysiology of DD in pediatric patients. In our pediatric patients supplemented with omega-3 FA, the plasma TXB levels were significantly reduced after 12 weeks, as compared to the omega-6 FA group. This could be explained by the fact that the omega-6/omega-3 FA ratio is reduced by the supplementation, [9] resulting in the displacement of omega-6 FA (arachidonic acid) from its pro-inflammatory effects.

Brain-derived neurotrophic factor (BDNF) is a protein that is implicated in the pathophysiology of depression. We did not find altered plasma BDNF levels in our pediatric patients, compared to healthy controls, contrary to the results of Pandey et al. [24], who found a decreased platelet BDNF gene and protein expression in depressed pediatric patients, compared to controls. The undetected difference in BDNF levels between DD patients and healthy children in our study at baseline could be explained by the antidepressant treatment of 37% of our patients before inclusion into the study, as antidepressants have been found to increase BDNF in blood [25]. However, we found the significantly reduced BDNF levels in depressed female patients, as compared to depressed male patients, to be in line with the results of Tsuchimine et al. [28]. This finding is consistent with our previous observation, where females had higher CDI scores (more severe symptoms of depression) than males at the baseline [9]. In addition, the role of BDNF in the pathophysiology of DD is supported by the finding of a significant negative correlation of BDNF with depression severity (CDI) (*p* ≤ 0.001) in our pediatric patients. In contrast to the findings of Pallavi et al. [45], who confirmed this correlation in pediatric males but not females, we have found a significant negative correlation of BDNF with CDI in both males (*p* = 0.014) and females (*p* = 0.009). We have also detected a negative correlation of BDNF with omega-6/omega-3 ratio (*p* = 0.038) in all patients. We realize that assessing the situation in the brain through systemic markers may not always be accurate. Similarly, the pathway of BDNF into the blood cannot be inferred from a human study, unless its two-way brain–blood transport is taken into account. BDNF synthesis is thought to be transcriptionally promoted by DHA [29]. However, we did not observe a significant correlation between BDNF and DHA or EPA, despite potential bidirectional transport of BDNF and possible transport of omega-3 FA across the blood–brain barrier (BBB) [46]. On the other hand, a negative association was observed between the depression severity (CDI) and EPA, but not DHA [9]. However, after dividing patients by diagnosis (DD subgroup and MADD), a significant correlation was found only between BDNF and EPA in the DD subgroup, but not with DHA, which similarly confirms the already published results [9]. The supplementation with omega-3 FA for 12 weeks significantly increased the plasma BDNF levels in all patients, compared to omega-6 FA intervention. It can be assumed that low concentrations of EPA and DHA contribute to the pathophysiology of DD in different ways. Among other things, the effectiveness of omega-3 FA supplementation depends on the accuracy of the diagnosis, age, EPA to DHA ratio, and the overall condition of the patients.

Homocysteine (HCy) is a non-proteinogenic sulfurated amino acid [47]. No differences in HCy levels in depressed adult patients and healthy individuals [47,48,49,50] or an association between HCy and DD were observed [47]. The increased risk of depression was reported only in older adults with a high HCy level [30]. Less information is available in depressed pediatric and adolescent patients. In our population, we did not observe an increased level of HCy or its correlation with CDI score or with the omega-6/omega3 ratio, contrary to the results of Esnafoglu and Ozturan [31], who found a significantly higher level of HCy and a positive correlation of HCy with the CDI score in depressed children and adolescents. Contrary to the observations of Chung et al. [32], who investigated anxiety in healthy students (boys 12 to 13 years old), we found a higher level of HCy in depressed males, compared to females (*p* < 0.001), but not in the healthy controls. Chung et al. [32] explained their observation in students through biological interactions between sex hormones in males and homocysteine [51]. However, the question remains why the observed differences in HCy levels in the males and females of our pediatric patients were not found also in our cohort of healthy controls. We did not find an association between depression and HCy in female or male patients. When we divided our pediatric patients by diagnosis (depressive disorder subgroup, DD-S), and mixed anxiety and depressive disorder, MADD), no statistical differences in HCy levels, nor the correlations between HCy and CDI or HCy and omega-6/omega-3 ratio were observed. The effect of omega-3 FA on HCy levels is discussed especially in adults, in association with cardiovascular and other diseases [52]. HCy is a metabolite of the C1 cycle and can be converted either to methionine by transmethylation in the presence of vitamin B12 and folate or to cysteine by trans-sulfuration in the presence of vitamin B6. In addition, it binds closely to lipids via betaine, which is formed from phosphatidylcholine upon release of choline. Betaine is then a cofactor for HCy methylation. In addition, other enzymes involved in the C1 cycle may be affected by omega-3 FA. For example, a precursor in HCy synthesis, S-adenosylhomocysteine inhibits phosphatidylcholine production and negatively correlates with DHA. However, other mechanisms are also possible [52]. However, the extent to which these relationships apply to children is not yet known.

The 12-week supplementation with the omega-3 FA or omega-6 FA did not have an impact on HCy levels. As our results do not lead to a clear conclusion regarding the association between HCy and the depression severity in children and adolescents, or the influence of gender, more studies with a well-defined diagnosis of DD are needed to confirm the findings.

Some observational studies highlighted an inverse association between serum vitamin D levels and depressive symptoms in adults [53]. On the contrary, the Mendelian randomization study on depression [37] and similar studies of Milaneschi et al. [38], Michaëlsson et al. [54] and Manson et al. [55]) indicate no association of vitamin D with depressive symptoms. However, the meta-analysis by Spedding [35] showed that the size effect of vitamin D on depression is comparable to the effect of antidepressants. The supplementation with vitamin D had a beneficial effect on the DD symptoms in 12 studies where the major depressive disorder diagnosis was established [56]. However, there is a lack of randomized, controlled trials in children and adolescents. In line with Libuda et al. [39] findings, we have observed the serum vitamin D deficiency (<20 ng/mL) in our group of depressed patients compared to healthy controls (*p* = 0.031), but our healthy control group also had an insufficient level of vitamin D (<30 ng/mL). Libuda et al. [39] did not confirmed a vitamin D effect on self-rated depression; however, the parents reported less significant depressive symptoms. Similarly, the vitamin D levels in our patients did not correlate with the severity of depression determined with self-rating CDI score or with omega-6/omega-3 ratio. Neither omega-3 FA nor omega-6 FA supplementation had any effect on vitamin D levels during the 12-week intervention. Both substances (omega-3 FA, vitamin D) affect the biosynthesis and serotonin levels and have an anti-inflammatory effect and some other common features [34]. However, their interactions are not fully known. In hemodialysis patients, Lee et al. [57] found that omega-3 FAs could affect the conversion of 25-hydroxyvitamin D to 1,25-dihydroxyvitamin D. However, our findings do not confirm a causal relationship between vitamin D and depressive symptoms in children and adolescents.

### Study Limitations

We did not determine any of the direct markers of inflammation (IL-6, IL-1, TNF, or others) in depressed children and adolescents, for technical reasons. Patients had hsCRP levels in the physiological range. We used an indirect marker, thromboxane, to indirectly monitor the inflammatory response.

The food diaries were not collected during the study. The patients were advised to maintain their normal diet and avoid the consumption of any fatty acid supplements or antioxidants throughout the study.

We are aware of a small number of males [9], as compared to females. Therefore, the gender assessment should be interpreted with caution.

## 5. Conclusions

Our findings suggest the potential involvement of thromboxane and brain-derived neurotrophic factor (BDNF) in the pathophysiology of depressive disorder in children and adolescents. A significant positive correlation was found between the depression severity or omega-6/omega-3 FA ratio and plasma thromboxane B and a negative correlation with BDNF. The children and adolescents with depressive disorders had higher levels of thromboxane B and reduced levels of vitamin D, when compared to healthy controls. The supplementation with omega-3 FA alongside the standard antidepressant therapy may have a beneficial effect on thromboxane levels. However, the positive effect of omega-3 FA supplementation on BDNF levels was seen only in depressed patients diagnosed with depressive disorder, but not in patients with mixed anxiety and depressive disorder (evaluated through a correlation between EPA, DHA, and BDNF). These findings are encouraging and need to be confirmed in a larger study of the pediatric population.

## Figures and Tables

**Table 1:** Baseline characteristics of depressed patients and healthy controls.

Parameter	Patients			*p-*Value	Healthy Controls			*p-*Value	*p-*Value
	All	Male	Female	M vs. F	All	Male	Female	M vs. F	P vs. C
*n*	58	12	46		20	8	12		
Age (years)	15.6 ± 1.6	16.4 ± 2.2	15.3 ± 1.3	0.235	14.8 ± 2.4	14.0 ± 2.5	14.4 ± 2.5	0.621	0.059
Weight (kg)	60.3 ± 11.7	68.2 ± 15.5	56.8 ± 9.3	**0.015**	54.9 ± 18.8	56.1 ± 21.2	54.1 ± 17.8	0.435	0.147
Height (m)	1.68 ± 0.1	1.74 ± 01	1.66 ± 0.1	**0.016**	1.6 ± 0.2	1.6 ± 0.2	1.6 ± 0.1	0.543	0.471
BMI (kg/m^2^)	21.14 ± 2.7	22.4 ± 3.6	20.5 ± 2.8	0.532	20.6 ± 4.2	20.1 ± 3.0	20.9 ± 5.0	0.498	0.537

M = male, F = female, P = patient, C = healthy controls, *p* = statistical significance, vs. = versus, *n* = number of individuals, the numbers in the bold are statistically significant.

**Table 2:** Baseline levels of TXB, BDNF, HCy, and vitamin D in all patients and healthy controls.

Parameter	All Patients	Healthy Controls	*p-*Value
	*n* = 58	*n* = 20	
TXB (pg/mL)	411.6 ± 288.7	151.2 ± 89.7	**<0.001**
BDNF (ng/mL)	631.8 ± 257.3	605.9 ± 95.9	0.678
HCy (µmol/L)	12.7 ± 8.1	11.6 ± 3.1	0.550
Vitamin D (ng/mL)	19.0 ± 7.4	23.1 ± 5.8	**0.031**

TXB = thromboxane B, BDNF = brain-derived neurotrophic factor, HCy = homocysteine, *p* = statistical significance, *n* = number of individuals in the group, the numbers in the bold are statistically significant.

**Table 3:** The effect of omega-3 and omega-6 FA on TXB, BDNF, HCy, and vitamin D in patients with depressive disorder.

Parameter	Om3 Group (*n* = 29)			Om6 Group (*n* = 29)			*p*-Values between Om3 and Om6 Groups	
	Week 6	Week 12	*p*-Value to Baseline *	Week 6	Week 12	*p*-Value to Baseline *	Week 6	Week 12
TXB (pg/mL)	318.3 ± 173.7	291.7 ± 140.0	**0.** **037**	425.9 ± 275.5	406.3 ± 180.1	ns	0.091	**0.024**
BDNF (ng/mL)	827.2 ± 441.3	824.7 ± 416.7	**0.040**	683.4 ± 413.9	592.5 ± 220.2	ns	ns	**0.011**
HCy (µmol/L)	13.6 ± 9.3	11.6 ± 8.1	ns	11.4 ± 5.7	11.3 ± 7.2	ns	ns	ns
Vitamin D (ng/mL)	18.7 ± 7.8	20.9 ± 8.3	ns	17.8 ± 5.2	21.7 ± 8.5	ns	ns	ns

TXB = thromboxane B, BDNF = brain-derived neurotrophic factor, HCy = homocysteine, *p* = statistical significance; * significance after 12 weeks of supplementation; ns = not significant, *n* = number of individuals in the group, the numbers in the bold are statistically significant.

**Table 4:** Correlations between parameters in patients and controls at baseline.

**All Patients**	***n***	***r***	***p***
CDI vs. TXB	56	0.411	**<0.001**
CDI vs. BDNF	52	−0.5	**<0.001**
CDI vs. HCy	58	−0.2	0.081
CDI vs. vitamin D	56	0.036	0.396
**All patients**	***n***	***r***	***p***
omega-6/omega-3 vs. TXB	56	0.304	**0.03**
omega-6/omega-3 vs. BDNF	52	−0.258	**0.038**
omega-6/omega-3 vs. HCy	58	0.08	0.282
omega-6/omega-3 vs. vitamin D	56	−0.088	0.269
**Healthy Controls**	***n***	***r***	***p***
omega-6/omega-3 vs. TXB	19	0.520	**0.01**
omega-6/omega-3 vs. BDNF	19	0.174	0.471
omega-6/omega-3 vs. HCy	19	0.058	0.099
omega-6/omega-3 vs. vitamin D	18	−0.028	0.915

CDI = Children’s Depression Inventory, TXB = thromboxane B, BDNF = brain-derived neurotrophic factor, HCy = homocysteine, vs. = versus, *n* = number of subjects, *r* = Spearman’s rank correlation coefficient, *p* = statistical significance, the numbers in the bold are statistically significant.

## Data Availability

The datasets generating and analyzed during the current study are not publicly available, due to ethical reasons, but are available from the corresponding author upon a reasonable request.

## Supplementary Materials

The following are available online at https://www.mdpi.com/article/10.3390/nu13041095/s1, Figure S1: Consort flow diagram.
